# Therapeutic outcomes of non-grafted and platelet concentrations-grafted transcrestal maxillary sinus elevation (TSFE): a systematic review and meta-analysis

**DOI:** 10.1038/s41598-020-62407-y

**Published:** 2020-04-03

**Authors:** Tianqi Guo, Karan Gulati, Ziyun Shen, Pingping Han, Zhen Fan

**Affiliations:** 1 The University of Queensland, School of Dentistry, Herston, QLD 4006 Australia; 2 Shanghai Engineering Research Center of Tooth Restoration and Regeneration, Shanghai, 200072 PR China; 3 Department of Oral Implant, School and Hospital of Stomatology, Tongji University, Shanghai, 200072 PR China

**Keywords:** Outcomes research, Dental implants, Maxillofacial surgery, Oral surgery

## Abstract

To evaluate and compare the stability, quantity and quality of bone augmentation at maxillary sinus elevation sites by non-grafted transcrestal sinus floor elevation (TSFE) and platelet concentration grafted transcrestal sinus floor elevation (PC-TSFE). A complete literature search was performed up to April 2019. Clinical controlled trials, retrospective cohort studies, and prospective cohort studies were selected based on inclusion criteria. The clinical outcomes were implant survival rate (ISR), marginal/crestal bone loss (MBL/CBL) and endo-sinus bone gain (ESBG). Meta‐analysis was conducted on these 1-year based values. Furthermore, another meta-analysis on 1-year ISR value was conducted among studies with different residual bone heights (RBH) within the non-grafted TSFE group. A total of 18 studies were included: 13 in TSFE group and 5 in PC-TSFE group. No significant differences were displayed between the 1-year ISR of TSFE (97%, 95%CI = 0.96–0.99) and PC-TSFE group (99%, 95%CI = 0.97–1.00). Among the various studies with different RBH within TSFE group, no significant differences in 1-year ISR were displayed. The 1-year MBL/CBL value of PC-TSFE group (0.73 mm, 95%CI = 0.43–1.13 mm) did not show significant difference as compared to TSFE group (0.60 mm, 95%CI = 0.10–1.10 mm). Furthermore, no significant enhancement was observed on 1-year ESBG value on PC-TSFE group (3.51 mm, 95%CI = 2.31–4.71 mm) in comparison with the TSFE group (2.87 mm, 95%CI = 2.18m–3.55 mm). Grafting platelet concentrations around dental implants at TSFE sites did not significantly enhance the adjacent bone regeneration. Moreover, TSFE was shown to be a reliable therapeutic option for implant sites that need simultaneous maxillary sinus augmentation, even under limited RBH.

## Introduction

The maxillary sinus is a cavity of pyramid shape in the maxilla with a volume of 12–15 mL. Its anterior border extends into the premolar roots or distal surface of canine roots, and the posterior border reaches the maxillary tuberosity^1,2^. Due to its structure and location, the maxillary sinus sometimes challenges the proper placement of the implant and also compromises its long-term functioning^2^.

To address this challenge and establish an adequate bone site for implantation, direct (lateral approach) maxillary sinus lift has been suggested since its first introduction in the 1980s^3^. That lateral approach is reliable to augment large quantity of bone at surgical sites, however it has strict limitations including excessive surgical trauma and prolonged healing time, which must be addressed^3^. In that attempt, transcrestal maxillary sinus elevation (TSFE), which is proceeded via alveolar crest, has been suggested and well implemented in clinical dentistry^4^. It compresses and apically pushes the maxillary bone from alveolar crest, and thus elevates maxillary sinus membrane for bone substitutes and implants^4^. The TSFE enhances the bone density around implant surfaces by compressing the alveolar bone, thereby providing the implant with enhanced primary stability^5–8^.

Although the TSFE is easy to handle with reduced surgical trauma compared with lateral maxillary sinus lift^5,6^, it has few limitations including inability to directly visualize membrane augmentation procedures and increased risk of membrane perforations on the TSFE sites. Furthermore, it is also unsuitable for cases with severely compromised bone quantity (RBH < 4 mm)^5,6^. RBH is one of the critical factors, together with bone quality/density, general health conditions, alignment of adjacent teeth and others, which determine the selection of implant surgery modality^2^. For cases with limited RBH (<8 mm), applying short implant or tilted implantation are two plausible options, however the optimized treatment modality is still TSFE, which significantly increases the available bone quantity and thus guarantee a sufficient osseointegration region^9^.

Various clinical studies have reported that TSFE on cases with limited RBH (4–6 mm) leads to favorable implant survival rates, as long as these cases can attain secured primary stability^7,8^, however the lateral/direct access of MSFA still remains as a “gold standard” for such cases with severely compromised bone quantity. This is attributed to limited RBH resulting in unsecured primary stability around the implants, and the inability of TSFE to provide direct visualization of membrane augmentation, thus increasing the risk of membrane penetration during surgery^6,10^. As is indicated by previous studies, grafting bone substitutions is not essential for a successful TSFE, mainly because implants and surrounding bone usually protrude into the maxillary sinus, which in turn hold the Schneiderian membrane and maintains a stable chamber for osteogenesis^11^. Furthermore, bone grafts and sharp tip of the implants inserted into maxillary sinus may penetrate the Schneiderian membrane and result in compromised bone healing^4,11^. However, research gaps remain unaddressed including lack of investigations to find an appropriate osteoinductive bone substitutions, which is both soft and flexible (acts like buffer to protect the fragile Schneiderian membrane) into maxillary sinus in TSFE sites.

Platelet concentrations are autologous plasma portion that are extracted from the autologous whole blood and enriched with cytokines to promote tissue regeneration^12–15^. Previous studies have indicated that platelet concentrations include platelet-rich plasma (PRP), plate-rich fibrin (PRF), platelet-rich growth factor (PRGF) and concentrated growth factor (CGF), etc.^12–15^. Currently, these varied platelet concentrations are clinically applied to promote wound healing, accelerate bone regeneration and modulate the post-surgical inflammation^16,17^. Although there are some deviations among these products, such as components and amount of cytokines, these concentrations are mechanically flexible (either in liquid or gel-like phase) and able to promote wound healing at surgical sites^12,13^. Moreover, the interlocking mesh-like microstructure created by the aggregation of platelets make the platelet grafts effective in promoting the migration and proliferation of osteoprogenitor cells and thus can enhance the osseointegration at the implant-bone interface^14^. Based on the fact that platelet concentrations are soft and contain numerous cytokines to promote bone regeneration, they have been reported as grafting materials into maxillary sinus lift sites by many studies^7,18–21^.

The aim of this study is to explore the therapeutic outcomes between non-grafted and platelet concentrations grafted TSFE. The vertical quantity and stability of newly regenerated bone in both grafted and non-grafted groups were evaluated by analyzing their implant survival rate, marginal bone loss (MBL) and endo-sinus bone gain (ESBG). Meanwhile, to evaluate the clinical reliability of TSFE under limited RBH, the implant survival rates among subgroups of different average RBH within TSFE group were also investigated. In summary, this study will provide insight into the clinical application of platelet concentrations as grafting materials in TSFE sites, and the therapeutic reliability of non-grafted TSFE in sites with poor bone quantity.

## Results

### Literature search and selection

**Figure 1:**
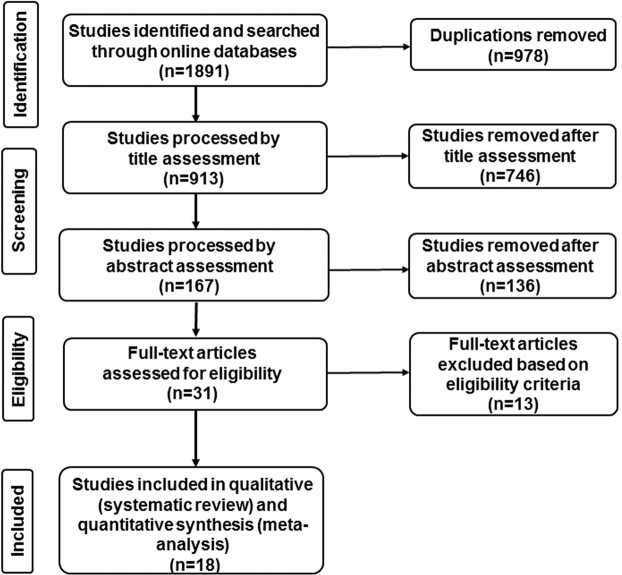
Flow diagram depicting the literature identification and selection in the current study.

The process for searching and selecting literature is shown in Fig. 1. A total of 1891 studies were searched and saved in Endnote X9 (n = 1891). After 978 duplications were removed, title of residual literatures (n = 913) were scanned and identified, which led to removal of 746, which did not fulfil the inclusion criteria. Then abstract assessments were performed on the remaining studies (n = 167), from which 136 studies were excluded. A final full-text reading was conducted on the remaining studies (n = 31). Furthermore, a total of 13 articles were excluded for the following reasons: 5 for repeat with subsequent studies^22–26^, 3 for inconsistent follow-up visit time^27–29^, 3 for the inaccurate and insufficient data^30–32^, 1 where grafted material did not fulfil the inclusion criteria^33^, and 1 was excluded for the inaccurate demographic data^34^. Finally, 18 studies were included in this systematic review and meta-analysis, among which were 13 non-grafted TSFE^35–47^, 5 TSFE grafted with various platelet concentrations (PRP, PRF, CGF or PRGF)^7,18–21^.

### Literature quality assessments

Tables 1 and 2 shows the literature quality assessments of the 3 controlled studies and 15 non-randomized studies. For the controlled studies, the literature reported by Si *et al*. in 2013 qualified as low risk of bias, while the studies by Lai H C (2010) and Nedir (2017) were regarded with moderate risk of bias. For the non-randomized studies (prospective and retrospective cohort studies), all the 15 studies scored 7 or more and were assessed as high quality with low risk of bias.

**Table 1:** The literature quality and risk of bias assessment of controlled studies by Cochrane scale form.

Author (Year)	Adequate sequence generation	Allocation concealment	Blinding of participants and personnel	Blinding of outcome assessment	Incomplete outcome data addressed	Selective outcome reporting	Free from other bias	The estimated risk potential of bias
Si M (2013)	Yes	Yes	Yes	Yes	Yes	Yes	Yes	Low
Lai H (2010)	No	Yes	No	No	Yes	Yes	Yes	Moderate
Nedir (2017)	Yes	Yes	No	No	Yes	Yes	Yes	Moderate

**Table 2:** The literature quality and risk of bias assessment of non-randomized studies by Newcastle-Ottawa scale form.

Author (Year)	Selection				Comparability	Outcome			Total
	A representative of the exposed cohort	Selection of external control	Ascertainment of exposure	Outcomes not present at the start	Comparability on design or analysis	Outcome assessment	Enough follow-up visit time	Adequacy of follow-up of cohorts	
Diss A (2008)	★	0	★	★	★0	★	★	★	7/9
Schleier P (2008)	★	0	★	★	★0	★	★	★	7/9
Siovio T (2011)	★	0	★	★	★★	★	★	★	8/9
Fermergard (2012)	★	0	★	★	★0	★	★	★	8/9
He L(2012)	★	0	★	★	★★	★	0	★	7/9
Vople S (2013)	★	0	★	★	★0	★	★	★	7/9
Brizeula A (2014)	★	0	★	★	★★	★	★	★	8/9
Kim J M (2014)	★	0	★	★	★0	★	★	★	7/9
Stiovio T (2014)	★	0	★	★	★0	★	★	★	7/9
Anitua.E (2015)	★	0	★	★	★★	0	★	★	7/9
Spinelli.D (2015)	★	0	★	★	★★	★	★	★	8/9
Gu Y X (2016)	★	0	★	★	★0	★	★	★	7/9
Nedir R (2016)	★	0	★	★	★★	★	★	★	8/9
Si M S (2016)	★	0	★	★	★0	★	★	★	7/9
Caban J (2017)	★	0	★	★	★0	★	★	★	7/9

### Demographics and surgical methods

As is presented in Table 3, among the 13 studies on non-grafted TSFE, Straumann implants were applied in 6 studies; Astra-tech implants were applied in 3 studies; 2 used both Neoss and Nobel Biocare implant systems; and 1 study used both Osstem and Bego implant systems. Furthermore, 1 study by Lai *et al*. did not clearly describe the implants’ brand name. There were 6 retrospective studies, 4 prospective studies, 2 randomized controlled tests and 1 clinical controlled test for the non-grafted TSFE group. All the 3 controlled tests compared the therapeutic outcomes of non-grafted TSFE with TSFE grafted with bone substitutions, and for these studies the data of the non-grafted group were extracted and analyzed in the meta-analysis. 5 studies had reported the platelet-grafted TSFE **(**Table 3), among which were 2 retrospective studies, 2 prospective studies, while the other 1 was not clearly defined. Considering the various grafted platelet concentrations, 2 of them were PRGF, the other 3 were PRP, PRF and CGF, respectively. 2 studies applied BTI implant system, 2 studies applied Zimmer and Astra-tech implants, respectively, and the other 1 was not clearly indicated.

**Table 3:** Demographic data and therapeutic outcomes of studies included in the final systematic review and meta-analysis.

**Non-Grafted TSFE**										
Author (year)	Implant brand	Implant numbers	Patient numbers	Surgical methods	RBH	Follow-up time	Implant survival rate	MBL/CBL	ESBG	Study category
Caban J(2017)	Astra Tech	34	25	TSFE	4.3 ± 1.0 mm	12	91.2%	0.4 ± 0.42	N/A	Retrospective
						60	91.2%	0.5 ± 0.45		
						120	91.2%	0.6 ± 0.82		
Nedir (2017)	Straumann AG	17	9	TSFE	2.4 ± 0.9	12	100%	0.6 ± 0.8	3.9 ± 1.0	Randomized controlled study
						36	94.12%	0.6 ± 1.1	4.1 ± 1.0	
						60	94.12%	0.6 ± 0.9	3.8 ± 1.0	
Nedir (2016)	Straumann AG	25	17	TSFE	5.4 ± 2.3	12	100%	1.2 ± 0.7	2.5 ± 1.2	Prospective
						36	100%	0.9 ± 0.8	3.1 ± 1.5	
						60	100%	0.8 ± 0.8	3.2 ± 1.3	
						120	100%	1.0 ± 0.9	3.0 ± 1.4	
Si M S (2016)	Straumann	96	80	TSFE	6.75 ± 1.91 mm	12	96.2%	N/A	N/A	Retrospective
						24	94.8%			
						36	92.7%			
						48	92.7%	0.46 ± 0.88	2.95 ± 1.25	
						60	90.6%	0.50 ± 0.96	3.01 ± 1.36	
						72	90.6%	0.50 ± 0.97	3.74 ± 1.34	
						84	90.6%	0.46 ± 1.08	2.63 ± 1.36	
						96	90.6%	0.48 ± 1.32	2.55 ± 1.11	
						108	90.6%	0.50 ± 1.69	2.16 ± 1.13	
					>=5 mm	108	93.5% (n = 72)	N/A	2.89 ± 1.16	Retrospective
					<5 mm		78.9% (n = 15)		3.24 ± 1.63	
Gu Y X (2016)	Straumann	37	25	TSFE	2.81 ± 0.74	12	94.6%	0.83 ± 0.50	N/A	Prospective
						36	94.6%	1.47 ± 1.02		
						60	94.6%	1.54 ± 1.00		
D.Spinelli (2015)	NobelSpeedy NobelActive	66	39	Template guided TSFE	6.7 ± 1.6	12	98.83%	0.33 ± 0.36	N/A	Prospective
						36	98.83%	0.51 ± 0.29	6.4 ± 1.6	
Aritza B (2014)	Straumann Klockner	36	N/A	TSFE	7.4 ± 0.4	24	91.6%	0.7 ± 0.1	1.8 ± 0.3	Prospective
Si M S(2013)	Straumann SLA	20	20	TSFE	4.58 ± 1.47	12	100%	1.28 ± 0.05	2.56 ± 0.98	Randomized control test
						36	95.0%	1.38 ± 0.23	3.07 ± 1.68	
Stefano V (2013)	Neoss Ltd Harrogate	29	20	TSFE	7.2 ± 1.5	11–32	100%	0.7 ± 0.3	2.8 ± 1.2	Retrospective
He L (2013)	Bego Osstem	27	22	TSFE	6.7 ± 1.2	6	100%	N/A	2.5 ± 1.5	Retrospective
Robert F (2012)	Astra	53	36	TSFE	6.3 ± 0.3	12	96%	0.5 ± 0.06	N/A	Retrospective
						36	94%	0.6 ± 0.09		
Lai H C (2010)	N/A	191	125	TSFE	5.6 ± 2.5	60	97.38%	N/A	N/A	Randomized control test
Peter S (2008)	Straumann SP Straumann TE	62	30	Endocsope guided TSFE	8.4 ± 2.2	24	94%	N/A	3.5 ± 1.8	Retrospective
**Platelet Concentrations Grafted TSFE**										
**Author (year)**	**Implant brand**	**Implant numbers**	**Patient numbers**	**Surgical methods**	**Residual bone height**	**Follow-up time**	**Implant survival rate**	**Marginal bone loss**	**ESBG**	**Study category**
E. Anitua (2015)	N/A	61	48	TSFE + PRGF	4.03 ± 0.51	12	98.3%	0.86 ± 0.49 (n = 30)	4.64 ± 1.68	Retrospective
Siovio T (2014)	BTI	65	25	TSFE + PRP	5.8 + 1.10	12	100%	0.35 ± 0.25	2.7 ± 1.29	Prospective
Ji-Min Kim (2014)	Zimmer	16	11	CGF + HPISE	4.98 ± 2.8	14	100%	N/A	8.23 ± 2.88	Retrospective
Siovio (2011)	BTI	15	15	TSFE + PRGF	N/A	24–50 (average 35.6)	100%	0.36 ± 0.19	2.9 ± 0,8	N/A
Diss A (2008)	Astra tech	35	20	TSFE + PRF	6.5 ± 1.7	12	97.1%	N/A	3.2 ± 1.5	Prospective

TSFE: transcrestal sinus floor elevation; RBH: residual bone height; MBL/CBL: marginal/crestal bone loss; ESBG: endo-sinus bone gain.

### Implant survival rate

**Figure 2:**
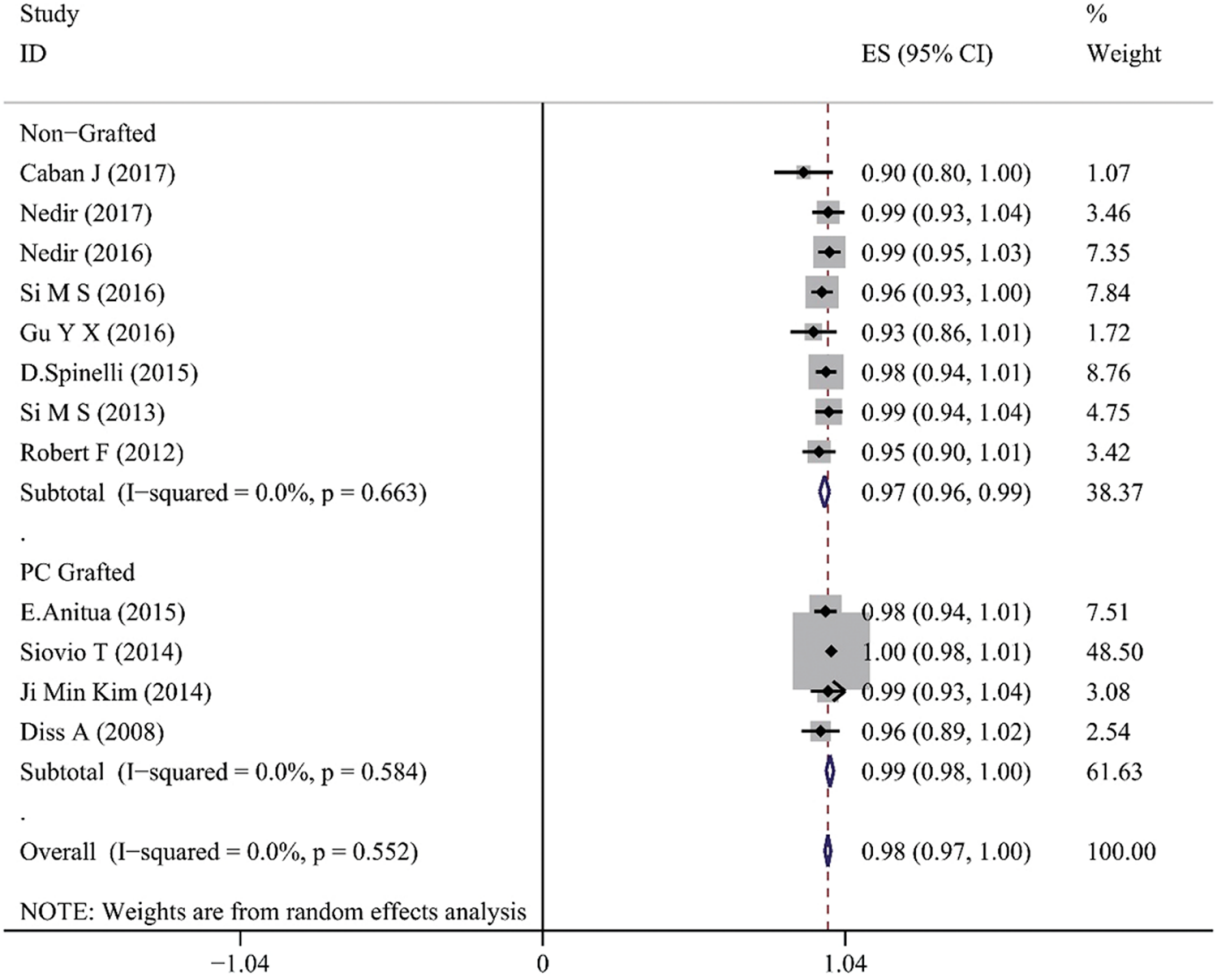
The meta-analysis on 1-year implant survival rate for the non-grafted TSFE and the PC-TSFE. Forest plot was generated by Stata 14.0, and randomized model was applied for the meta-analysis. PC: platelet concentrations. ES: average value for implant survival rate. CI: confidential interval.

**Figure 3:**
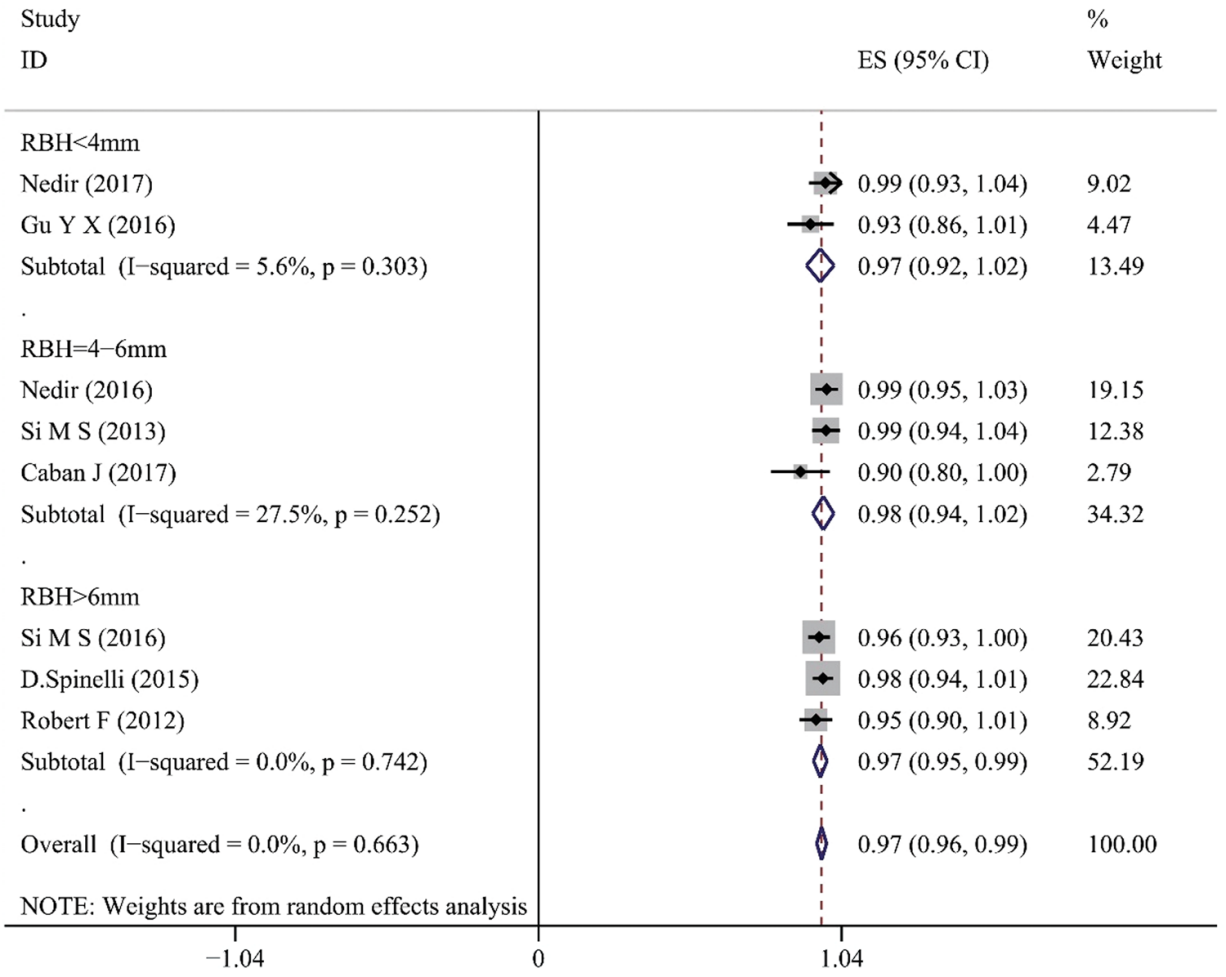
The meta-analysis on 1-year implant survival rate among subgroups with varied RBH within non-grafted TSFE. Forest plot was generated by Stata 14.0, randomized model was applied for the meta-analysis. RBH: residual bone height; TSFE: transcrestal sinus floor elevation. ES: average value for implant survival rate; CI: confidential interval.

As presented in Table 3, for the non-grafted TSFE group, the cumulated implant survival rates ranged between 90.6% and 100%, within the varied follow-up visit time (6–120 months). For the studies with longest follow-up visit time (10 years), the 10-year cumulative implant survival rate was 100% in Nedir’s study (25 implants), and 91.2% in Caban’s study (31/34 implants, 3 failed implants occurred within the 1^st^ year)^35,36^. The lowest implant survival rate was 90.6% (87 out of 96 implants survived) over 108 months, as is reported by Si *et al*.^38^. For the platelet-grafted TSFE, 4 out of 5 studies reported their 1-year implant survival rates, which ranged between 97.1% and 100%^7,18–20^. The only failed implant occurred in the study by Diss A *et al*., which reported 1 out of 35 implants failed at 1 year after treatment with PRF-grafted TSFE^21^. The meta-analysis on the 1-year implant-based survival rate of non-grafted TSFE showed an average value of 97% (95%CI = 0.96–0.99), while the PC-TSFE was 99% (95%CI = 0.97–1.00) (Fig. 2). Although slightly higher average ISR was obtained in the PC-TSFE group, the non-grafted TSFE group still obtained a favorable 1-year ISR, which indicated that it is a reliable treatment modality for augmenting bone quantity in maxillary edentulous region. Furthermore, another meta-analysis on 1-year implant-based survival rate among different RBH within non-grafted TSFE studies indicated that the survival rate for studies with RBH < 4 mm was 97% (95%CI 0.92–1.00), for whose RBH = 4~6 mm was 98% (95%CI 0.94–1.00), and those with RBH > 6 mm was 97% (95%CI 0.95–0.99) (Fig. 3). Finally, all the subgroups obtained favorable 1-year ISR values, which indicated that insufficient RBH is not restriction for non-grafted TSFE on maxillary edentulous region.

### Marginal bone loss

**Figure 4:**
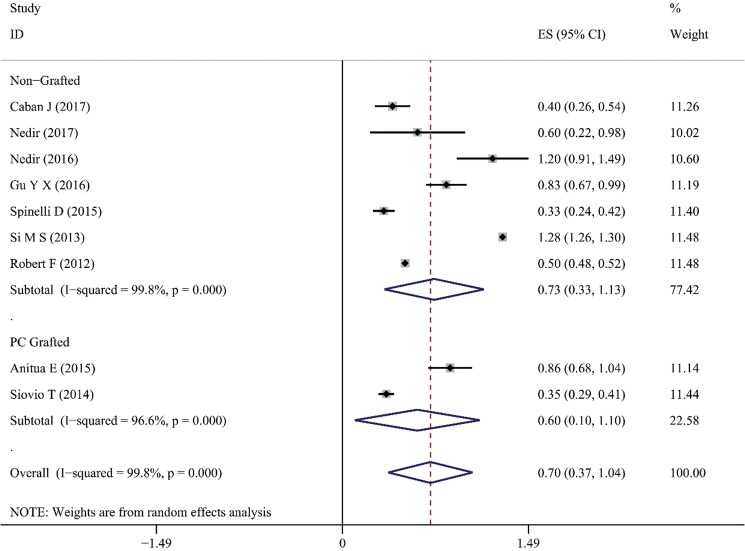
Result of meta-analysis on 1-year marginal bone loss (MBL) for non-grafted and PC-TSFE groups. Forest plot was generated by Stata 14.0, randomized model was applied for the meta-analysis. PC: platelet concentrations. ES: average value for 1-year MBL; CI: confidential interval.

1-year marginal bone loss (MBL) has been reported by 7 studies in non-grafted TSFE, with the majority of them (5 out of 7) being less than 1 mm. Spinelli *et al*. and Fermergrad *et al*. reported the smallest 1-year MBL, which were 0.33 ± 0.36mm and 0.5 ± 0.06mm, respectively^26,40^. While the largest 1-year MBL occurred in the study by Si *et al*. (1.28 ± 0.05 mm) and Nedir *et al*. (1.2 ± 0.7 mm)^36,38^. As presented in Table 3, the majority of MBL value remains stable 1-year post-surgery, except the study reported by Gu *et al*.^39^. For the 5 studies which reported long-term MBL (≥5 years), the majority of them reported an average 5-year MBL that was less than 1 mm except for Gu’s study (1.54 ± 1.00 mm). 2 studies in the PC-TSFE group had reported the average 1-year MBL: (1) Siovio’s *et al*. reported an average 1-year MBL of 0.35 ± 0.25 mm, and (2) Anitua *et al*. reported an average 1-year MBL of 0.86 ± 0.49 mm^7,18,19^. Meta-analysis on 1-year MBL indicated that the group of non-grafted TSFE was 0.73 mm (95%CI 0.43 mm–1.13 mm), while for the PC-TSFE was 0.60 mm (95%CI 0.10 mm–1.10 mm) **(**Fig. 4**)**. Calculations from the equation revealed changes of 1-year MBL value between non-grafted TSFE and PC-TSFE to be 0.13 ± 3.36 mm, and since 0 was contained in this 95% CI (−0.21 mm, 0.47 mm),hence no significant differences were obtained between the 1-year MBL of PC-TSFE and non-grafted TSFE groups.

### Endo-sinus bone gain (ESBG)

**Figure 5:**
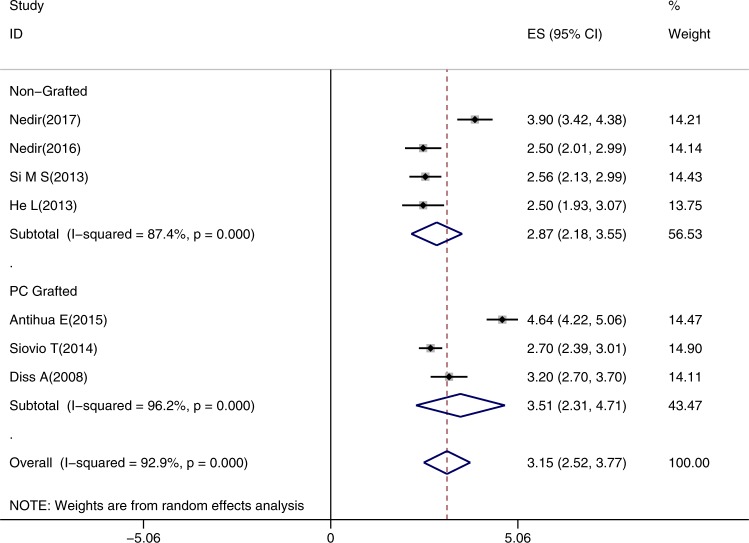
Result of meta-analysis on 1-year endo-sinus bone gain (ESBG) between non-grafted and PC-TSFE groups. Forest plot was generated by Stata 14.0, randomized model was applied for the meta-analysis. PC: platelet concentrations. ES: average value for 1-year ESBG; CI: confidential interval.

8 out of 12 studies on non-grafted TSFE reported ESBG after surgery. The lowest ESBG occurred in Aritza’s study, which was 1.8 ± 0.3 mm over 24 months, while the highest value was reported by Spinell, which was 6.4 ± 1.6 mm over 36 months^40,41^. 4 studies reported the change of ESBG value during a sequential follow-up visit time, which showed to be stable 1-year post-surgery, except Si’s study which showed a significantly enhanced ESBG at 3 year as compared to of 1 year^42^. In the PC-TSFE group, all 5 studies reported the postsurgical ESBG, and the highest value was 8.23 ± 2.88 mm by Kim *et al*. at 14-months post-surgery^20^. The meta-analysis on 1-year ESBG includes 3 studies, which excluded the study by Siovio and Kim *et al*.^40–42^. The results confirm that average 1-year ESBG was 2.87 mm for non-grafted TSFE (95%CI 2.18m-3.55 mm), and 3.51 mm for the PC-TSFE (95%CI 2.31–4.71 mm) **(**Fig. 5**)**. As per the equation, changes of 1-year ESBG between non-grafted TSFE and PC-TSFE was 0.64 ± 7.25 mm, and since 0 was contained within its 95% CI (−0.15 mm, 1.43 mm), no significant enhancements were observedon ESBG value after the intervention (applying PC in TSFE sites).

## Discussion

The chamber of augmented maxillary sinus created by the apical displacement of maxillary sinus membrane (Schneiderian membrane) has active osteogenic potential^48,49^. As previous study indicated, cells extracted from the Schneiderian membrane can express osteogenic differentiation cytokines including CD105, CD73 and CD166^48^. Moreover, there also are numerous ALP and STRO-I positive staining cells that can be separated from the Schneiderian membrane, which revealed the existence of osteoprogenitor cells and osteoblasts^49^. Based on these facts, it is indicated that bone grafts are not mandatory for a successful MSFA, as long as we could maintain the integrity of Schneiderian membrane and obtain a stabilized chamber for bone regeneration^50^. Studies focused on non-grafted TSFE have been performed and confirmed that such technique is reliable for MSFA sites, even in cases with limited residual bone height (RBH)^51^. Considering the longevity of non-grafted TSFE, some studies have even shown that there were no differences in implant survival rates between bone substitutions grafted and non-grafted TSFE sites^8,11^. However, for the lateral MSFA, bone grafts are still recommended as a beneficial strategy, for both improving the implant survival rates and maintaining the stability of ESBG^52^.

Platelet concentrations (PCs) are extracted by centrifuging autologous blood and formed by the co-aggregate process of platelets, and they could be fabricated into the liquid form including PRP, PRGF or gel-like substrates such as PRF and CGF^9,53–55^. All these concentrations are rich in various cytokines that promote tissue regeneration, which include vascular endothelial growth factor (VEGF), insulin growth factor (IGF) and platelet-derived growth factor (PDGF)^12–15,56–59^. The release of cytokines from platelet concentrations is effective towards upregulating osteoblast proliferation and regeneration of periodontal ligament cells^59^. Furthermore, the slow platelets aggregation allows for more cytokines to be embedded within such mesh-like structures, and their slow degradation rates allows for sustained release of growth factors into the surrounding tissue^12^.

Another distinguished feature of platelet concentrations is their softness and flexibility, which can act like a buffer material between protruded bone fragments/implant screws and Schneiderian membrane^9,53–55^. Compared with the traditional bone grafts that contain particles with sharp edges, the liquid/gel-like substrates of platelet concentrations are protective to the delicate Schneiderian membrane from being penetrated^9,53–55^.

In this study, initially we analyzed the implant survival rate (ISR) within non-grafted TSFE studies among different RBH, and no significant differences were observed on 1-year ISR among groups with different average RBH within non-grafted TSFE group, even cases with limited RBH (<4 mm) obtained favorable 1-year ISR^51,60,61^. This confirmed that limited RBH does not impact the implant survival for non-grafted TSFE surgeries, as long as secured primary stabilities are obtained on implants^51,60,61^. Furthermore, the increase in bone-implant contact area and the compression of bone during TSFE augments the bone density around implant sites. This in turn can enhance the primary stability of implant at TSFE sites, which effectively reduces the fretting of implants and promotes osseointegration at implant-bone interface^51^.

To investigate the enhancement of osseointegration and bone stability by adding PCs in TSFE sites, we performed meta-analysis on average 1-year ISR between non-grafted TSFE and PC-TSFE, which has not been reported before. Result showed no significant differences on 1-year ISR between the two groups, indicating that adding PCs in TSFE sites will not significantly enhance the strength and stability of osseointegration around implants. It is noteworthy that TSFE will press implant screws and adjacent bone into maxillary sinus, which are capable of supporting the apically displaced Schneiderian membrane, thus maintaining the stable osteogenic chamber^62^.

Furthermore, in the studies with MBL, there was no significant difference between the non-grafted TSFE and PC-TSFE groups. This can be attributed to the fact that PCs were lifted to the apical region of surgical sites, not the crestal region which could not directly influence the MBL during the constant masticatory cycles. It is noteworthy that MBL around implants is influenced by various factors including smoking, platform-switching technique at implant-abutment interfere, surface characteristics of implants/abutments, surgical trauma and functional loadings^63–65^. In the current study, the average 1-year MBL of non-grafted TSFE (0.73 mm, with 95%CI = 0.33–1.13 mm) was shown to be acceptable for the clinical application. Enhancement of peri-implant bone density alleviates the MBL, and the TSFE increases the density of peri-implant cancellous bone; hence the non-grafted TSFE can supports primary stability and peri-implant bone density (towards long term implant success)^66–68^.

For the ESBG data, although the PC-TSFE group showed a slightly higher 1-year average ESBG, there was no significant difference with non-grafted TSFE group. This is because PCs are flexible and gel-like substrates, which are not mechanically rigid to maintain extra augmentations of Schnederian membrane in TSFE sites. Furthermore, although PCs are are known to stimulate proliferation, migration and differentiation of adjacent osteoblasts/fibroblasts, their biodegradation characteristics does not continuously support the elevated Schneiderian membrane^69–71^. It is noteworthy that the chamber for bone regeneration at TSFE sites is maintained by the pressed implants and the displaced maxillary bone, thus the non-grafted TSFE is sufficient towards maintaining the vertical bone gain. These results correlates with the study by Liu *et al*., which reported that applying PCs as adjunctive to bone substitutions into MSFA sites did not show significant enhancement of regeneration and formation of new bone around implants^72^. Moreover, PCs will resorb within a few days after being implanted in human body. Such rapid degradation prevents them from constantly releasing cytokines during the bone regeneration phase in surgical sites^72^.

In summary, PCs contain various cytokines to promote bone regeneration. However, they are unable to significantly enhance the stability and vertical quantity of newly regenerated bone around the implant surfaces in TSFE sites. This is attributed to the inappropriate mechanical (softness and flexibility) and biodegradation characteristics of platelet concentrations, which prevents them from maintaining an osteogenic chamber by continuously supporting the Schneiderian membrane. Moreover, non-grafted TSFE is a reliable treatment modality for sites needing MSFA, even with limited RBH (<4 mm). To optimize their osteogenic potential, combining PCs with mechanically rigid and slow degrading bone substitutions to continuously support osteogenic chamber, can promote bone quantity and quality in TSFE sites. However, this represents a research gap and hence needs further investigations. Additionally, for further enhancement of PC-induced bone regeneration, more histological studies on bone formation pattern and process (including vascularizationand mineralization) are needed. Finally, the clinical studies on PC-TSFE are limited and most of them are based on short-term post-surgical data (mainly 1 year), which is a shortcoming for the current systematic review and requires long-term investigations for further advancements.

## Conclusion

This systematic review and meta-analysis compared the therapeutic outcomes of non-grafted transcrestal sinus floor elevation (TSFE) with platelet concentrations grafted TSFE (PC-TSFE). The results indicated no significant enhancements on 1-year implant survival rates, marginal bone loss (MBL) and endosinus bone gain (ESBG) of PC-TSFE group as compared to non-grafted TSFE group. Moreover, among subgroups of different residual bone height (RBH) within the non-grafted TSFE group, no significant differences were shown on their implant survival rates. The results confirm that adding platelet concentrations into maxillary sinus could not provide significant enhancements to the therapeutic outcomes of TSFE. However, to provide strong evidence on the therapeutic outcomes non-grafted and platelet grafted TSFE long term clinical studies must be conducted. Finally, to utilize the osteogenic potential of platelet concentrations and obtain an optimized bone quality/quantity in TSFE sites, further clinical and histological studies are needed, specially with the combined graft of platelet concentrations with other bone substitutes into TSFE sites. To date, the non-grafted TSFE remains a reliable therapeutic option for surgical sites that need maxillary sinus floor elevation (MSFA), even in cases with limited RBH.

## Methods and materials

### Search strategies

The PICO elements for this study are P (Problem): insufficient bone quantity at maxillary molar sites; I (intervention/indicator): grafting platelet concentrations in TSFE sites; C (comparison): non-grafted TSFE surgeries; O (outcome of interest): enhanced bone regeneration and osseointegration. The initial literature search was conducted in the Scopus, Pubmed and Cochrane library database. All relevant studies until April 2019 were included relating to indirect/transcrestal maxillary sinus elevation (TSFE). A search strategy of Mesh term or Keywords included a combination of [“maxillary sinus floor augmentation (MSFA)” OR “maxillary sinus lift” OR “maxillary sinus augmentation” OR “maxillary sinus elevation”] and [“transcrestal” OR “osteotomy” OR “transcrestal sinus floor elevation (TSFE)” OR “indirect sinus floor elevation” OR “osteotomy sinus floor elevation (OSFE)”] (processed by TG and ZF). After removing the duplications, a sequential selection of title, abstract and full text reading were performed on the residual literatures and finally selected. These articles included the studies relating to non-grafted TSFE and PC-grafted TSFE. Search and selection of literature was conducted by two reviewers (TG and ZF). If disagreements occurred between these two reviewers, a third reviewer (ZS) was recommended to judge the disagreement. All the literature were managed by Endnote X9.

### Inclusion criteria

Randomized controlled trials (RCTs), controlled clinical trials and observational studies (prospective and retrospective cohort studies) on the studies related to non-grafted TSFE or PC-grafted TSFE were included in this study. Minimum implants enrolled in each study was 15, and the shortest post-surgical observation period was 6 months. Moreover, demographic data of implants and patients (brand of implants, surgical methods, implant and patient numbers) must be clearly described. For studies that compare the therapeutic outcomes between non-grafted TSFE and bone-substitutions grafted TSFE, the outcomes of the non-grafted groups were extracted and analyzed in this paper. For studies which compared PC-grafted TSFE with bone substitutions grafted TSFE, data of the PC-TSFE group were extracted and compared.

### Exclusion criteria

Case reports, technical reports, conference articles, animal experiments, *in vitro* studies, finite element analysis, reviews and letters to the editors were all excluded. Clinical studies with insufficient post-surgical follow-up visiting periods, or inaccurately described therapeutic outcomes, and those unable to clearly define demographics were also excluded.

### Study selection and data extraction

Study selections were conducted by two researchers (TG and ZF), and the detailed process is presented in Fig. 1. When disagreements occurred, a third person (ZS) was asked to review and decide. Data of each selected study was extracted by two reviewers (TG and ZF), including: (1) demographics: authors, year of publishing, implant systems, numbers of implants and patients; (2) individual information: surgical methods and residual bone height (RBH); and (3) therapeutic outcomes: implant survival rate (ISR), endo-sinus bone gain (ESBG) and crestal/marginal bone loss (CBL/MBL). For studies that reported the therapeutic outcomes in sequential follow-up visit time, the data at all follow-up visit time points were extracted into the systematic review.

### Literature quality assessment

Quality assessment on the selected literature was performed by two researchers (TG and FZ). As showed in Table 1, these controlled studies were assessed by eight criteria under the Cochrane Collaboration guidelines^73^. An assessed study was confined as “low risk of bias” if all the criteria were answered “Yes”. If 1–2 clauses were “No”, the risk bias was defined as “moderate”. If more than 2 clauses were “No”, the risk of bias was outlined as “high”. Newcastle-Ottawa Scale (NOS) was applied for assessing the quality of non-randomized studies (prospective and retrospective cohort studies), just as Table 2 describes, with the maximum possible score 9. Any study scored 7 or more was determined as high quality with low risk of bias^73^.

### Data synthesis and meta-analysis

Stata MP 14.1 software was applied to process the meta-analysis, randomized model was applied to process the meta-analysis, with heterogeneity indicated by I-square values. To include studies having a 100% implant survival rate, we modified the rates with the formula: [modified survival rate = (number of survived implants − 0.25)/(number of total implants) *100%] in Stata MP 14.1. Considering the data of ISR, MBL and ESBG were based on the 1-year follow-up in majority of the literature, the 1-year post-surgical data of these parameters were considered in the meta-analysis. Moreover, to investigate the clinical reliability of non-grafted TSFE in cases with limited RBH, study on 1-year ISR of non-grafted TSFE was performed among its subgroups with varied RBH values.

### Variables processing and comparison

Since the 1-year ISR values pooled by Stata 14.0 were only accurate to their single digital units (eg. 0.99 = 99%), thus observation and descriptions were applied on these pooled values. For exploring the differences of 1-year MBL and ESBG between non-grafted and PC-grafted TSFE, these data were pooled and compared as described below:

Stata 14.0 applies 95% confidence interval (CI) to display the distribution of continuous variables, as indicated by Cochrane Handbook for Systematic Reviews (Version 5.0.2 Chapter 7.7.3.2.), to calculate the standard deviation (SD) of each group from the 95%CI, the equation below was applied^74^:

$$SD=\sqrt{n}\times (upper\,limit-lower\,limit)/3.92$$

As grafting PC is the intervention (I) in this study, thus the changes of average value, SD and standard error (SE) of each variable after intervention were calculated as the equations below, indicated by Cochrane Handbook for Systematic Reviews (Version 5.0.2 Chapter 16.1.3.2.)^74^:

$$Mean\,(E,\,change)=Mean\,(PC)-Mean\,(Non)$$

$$SD\,(E,\,change)=\sqrt{SD{(PC)}^{2}+SD{(Non)}^{2}-2\times Corr\times SD(PC)\times SD(Non)}$$

$$SE(E,change)=\frac{SD(E,change)}{\sqrt{n}}$$

In the equations above, *Non* represents non-grafted TSFE group and *PC* represents PC-grafted TSFE group, and *Corr* value was determined as 0.40, if the 95% CI (Mean_(E, change)_ ± 1.96×SE_(E, change)_) of changes in any variables contain 0, it is indicated that such variable had no significant enhancements/differences after grafted by PC, which is the intervention (I).
